# Sustained Baclofen-Induced Activation of GABA_*B*_ Receptors After Cerebral Ischemia Restores Receptor Expression and Function and Limits Progressing Loss of Neurons

**DOI:** 10.3389/fnmol.2021.726133

**Published:** 2021-09-01

**Authors:** Mohammad Hleihil, Markus Vaas, Musadiq A. Bhat, Karthik Balakrishnan, Dietmar Benke

**Affiliations:** ^1^Institute of Pharmacology and Toxicology, University Zurich, Zurich, Switzerland; ^2^Neuroscience Center Zurich, ETH Zurich, University of Zurich, Zurich, Switzerland

**Keywords:** GABA_*B*_ receptor, cerebral ischemia, MCAO, OGD, baclofen, neuroprotection

## Abstract

One important function of GABA_*B*_ receptors is the control of neuronal activity to prevent overexcitation and thereby excitotoxic death, which is a hallmark of cerebral ischemia. Consequently, sustained activation of GABA_*B*_ receptors with the selective agonist baclofen provides neuroprotection in *in vitro* and *in vivo* models of cerebral ischemia. However, excitotoxic conditions severely downregulate the receptors, which would compromise the neuroprotective effectiveness of baclofen. On the other hand, recent work suggests that sustained activation of GABA_*B*_ receptors stabilizes receptor expression. Therefore, we addressed the question whether sustained activation of GABA_*B*_ receptors reduces downregulation of the receptor under excitotoxic conditions and thereby preserves GABA_*B*_ receptor-mediated inhibition. In cultured neurons subjected to oxygen and glucose deprivation (OGD), to mimic cerebral ischemia, GABA_*B*_ receptors were severely downregulated. Treatment of the cultures with baclofen after OGD restored GABA_*B*_ receptor expression and reduced loss of neurons. Restoration of GABA_*B*_ receptors was due to enhanced fast recycling of the receptors, which reduced OGD-induced sorting of the receptors to lysosomal degradation. Utilizing the middle cerebral artery occlusion (MCAO) mouse model of cerebral ischemia, we verified the severe downregulation of GABA_*B*_ receptors in the affected cortex and a partial restoration of the receptors after systemic injection of baclofen. Restored receptor expression recovered GABA_*B*_ receptor-mediated currents, normalized the enhanced neuronal excitability observed after MCAO and limited progressive loss of neurons. These results suggest that baclofen-induced restoration of GABA_*B*_ receptors provides the basis for the neuroprotective activity of baclofen after an ischemic insult. Since GABA_*B*_ receptors regulate multiple beneficial pathways, they are promising targets for a neuroprotective strategy in acute cerebral ischemia.

## Introduction

The main cause for progressing neuronal death in cerebral ischemia is excitotoxicity. Massive release of glutamate excessively stimulates glutamate receptors, resulting in neuronal overexcitation and apoptotic death (Lipton, 1999; Szydlowska and Tymianski, 2010; Kostandy, 2012; Bano and Ankarcrona, 2018; Choi, 2020). Under physiological conditions, the excitability of neurons is precisely controlled by G protein coupled GABA_*B*_ receptors (Chalifoux and Carter, 2011a). GABA_*B*_ receptors are assembled from two subunits, GABA_*B1*_ and GABA_*B2*_, and are abundantly expressed at pre- and postsynaptic areas of most neurons. Activation of GABA_*B*_ receptors by the neurotransmitter GABA provides long lasting tonic inhibition mainly by suppression of voltage gated Ca^2+^ channel activity and activation of inwardly rectifying K^+^ channels. This inhibits neurotransmitter release *via* GABA_*B*_ receptors located at presynaptic sites and reduces neuronal excitability *via* postsynaptic receptors (Gassmann and Bettler, 2012). One function of GABA_*B*_ receptors is to act as an “emergency brake” that prevents neurons from shifting into overexcitation and apoptotic cell death (Chalifoux and Carter, 2011a). However, under the strong excitotoxic conditions caused by cerebral ischemia, GABA_*B*_ receptors are downregulated (Kim et al., 2011; Zhu et al., 2015; Huang et al., 2017) and are apparently no longer able to protect neurons from overexcitation. Studies on cultured neurons revealed that downregulation of GABA_*B*_ receptors is primarily due to aberrant sorting of the receptors to lysosomal degradation on the expense of recycling them to the cell surface (Guetg et al., 2010; Maier et al., 2010; Terunuma et al., 2010; Kantamneni et al., 2014).

Most G protein coupled receptors (GPCRs) undergo arrestin-mediated internalization upon agonist stimulation, which involves phosphorylation *via* G protein receptor kinases and uncoupling the receptors from G proteins. This process stops GPCR signaling and provides protection against excessive receptor activity (Gurevich and Gurevich, 2019). In contrast, activation of GABA_*B*_ receptors appears to stabilize them at the cell surface. Although activation of GABA_*B*_ receptors accelerates their internalization (Wilkins et al., 2008; Zhang et al., 2015), this appears to be counterbalanced by their enhanced recycling to the cell surface (Grampp et al., 2008; Zhang et al., 2015). Agonist stimulated recycling of GABA_*B*_ receptors involves the recruitment of the small GTPase Rap1, which physically interacts with GABA_*B*_ receptors and promotes receptor recycling by an unknown mechanism (Zhang et al., 2015).

Since sustained activation of GABA_*B*_ receptors appears to stabilize receptor numbers on the cell surface, it might well be that this effect contributes to the neuroprotective activity of the selective GABA_*B*_ receptor agonist baclofen. Baclofen exhibit neuroprotection in *in vitro* and *in vivo* models of cerebral ischemia (Lal et al., 1995; Jackson-Friedman et al., 1997; Kulinskii and Mikhel’son, 2000; Babcock et al., 2002; Dave et al., 2005; Zhang et al., 2007; Xu et al., 2008; Cimarosti et al., 2009; Liu et al., 2015). This suggests that either sustained activation of remaining GABA_*B*_ receptors after the ischemic insult is sufficient to promote neuronal survival or, alternatively, that persistent activation of the receptors stabilizes them at the cell surface and reduces their downregulation, making the baclofen treatment more efficient.

In this study, we tested whether sustained activation of GABA_*B*_ receptors reduces their ischemia-induced downregulation using cultured neurons subjected to oxygen and glucose deprivation (OGD) and the middle cerebral artery occlusion (MCAO) mouse model of cerebral ischemia. We found that sustained activation of GABA_*B*_ receptors restored receptor expression by inhibiting the ischemia-induced aberrant sorting of the receptors to the lysosomal degradation pathway. This was due to enhanced fast recycling of the receptors. The re-established GABA_*B*_ receptor-mediated inhibition reduced neuronal excitability and limited progressing loss of neurons.

## Materials and Methods

### Antibodies and Drugs

The following primary antibodies were used in this study: Rabbit anti NeuN (1:400 for microscopy, 1:1000 for Odyssey Scanner, Millipore Cat# ABN78), mouse anti GABA_*B2*_ (1:400 for microscopy, abcam Cat# ab181736), rabbit anti GABA_*B2*_ (1:2000 for Odyssey Scanner, abcam Cat# ab75838), mouse anti EEA1 (1:500, BD Biosciences Cat# 610456), mouse anti Rab11 (1:100, Millipore Cat# 05-853), and goat anti Rab7 (1:50, Santa Cruz Cat# sc-11303). Secondary antibodies: donkey anti rabbit Alexa Fluor Plus 488 (1:2000, Thermo Fisher Scientific Cat# A32790), donkey anti mouse Alexa Fluor Plus 555 (1:2000, Thermo Fisher Scientific Cat# A32773), and goat anti rabbit Alexa Fluor Plus 800 (1:2000, Thermo Fisher Scientific Cat# A32735). All chemicals used for electrophysiology solutions were purchased from Sigma-Aldrich and baclofen was purchased from Tocris Bioscience.

### Animals

Animal experiments were performed according to the national guidelines of the Swiss Federal act on animal protection. Experimental conditions were approved by the Cantonal Veterinary Office Zurich, Zurich, Switzerland (license ZH152/16 and ZH011/19). MCAO experiments were performed using 9–12 weeks old C57BL/6J male mice purchased from ENVIGO (Netherlands). Mice were housed up to five per cage with a standard 12/12-h light/dark cycle. Food and water were available *ad libitum*. Primary neuron cultures were prepared from E18 embryos of time pregnant Wistar rats obtained from ENVIGO.

### Primary Neuron Culture

Primary co-cultures of neurons and glia cells were prepared from E18 Wistar rat embryos as described in Buerli et al. (2007). Cells dissociated from minced cerebral cortex tissue were plated at a density of 60,000 cells onto poly-D-lysine coated 15 mm diameter coverslips and incubated overnight in complete Dulbecco’s modified Eagle Medium (DMEM; Thermo Fisher) at 37°C and 5% CO_2_. Subsequently, medium was replaced by the MEM/Nu-Serum based growing medium and kept at 37°C and 5% CO_2_ for 12–16 days *in vitro* (DIV).

### Oxygen–Glucose Deprivation

Oxygen–glucose deprivation was carried out by replacing the Nu-based culture medium by OGD medium (DMEM free of glucose, glutamine, and phenol red) depleted from oxygen by bubbling nitrogen for 15 min through the medium in a water bath at 37°C. Coverslips containing the cells were incubated in OGD media in a hypoxic incubator (1% O_2_, 5% CO_2_, 37°C) for 1 h. After the incubation, cells were transferred back to the original conditioned medium and incubated at 37°C and 5% CO_2_ until further use.

### Middle Cerebral Artery Occlusion

Transient MCAO was conducted using C57Bl/6J naïve male mice as previously described (Vaas et al., 2017). Before the surgery, mice were injected with buprenorphine (0.1 mg/kg, s.c.) and with lidocaine (5 mg/kg, s.c.) at the site of incision. The mice were anesthetized with 5% isoflurane and maintained at 1.5–2% isoflurane in a mixture of O_2_ and air during the surgery. The left common carotid artery was exposed through a midline incision in the neck and unilateral MCAO was conducted by inserting a 7-0 silicone rubber-coated monofilament to occlude the middle cerebral artery (Catalog No.: 701956PK5Re, Doccol Corp., Sharon, MA, United States). The middle cerebral artery was occluded for 60 min and then the filament was withdrawn to allow reperfusion. Following the surgery, mice were transferred to a 37°C chamber for recovery before moved back to the home cage. For sham operated mice, the surgery was identical to the animals undergoing MCAO except that the filament was inserted to occlude left MCA and immediately withdrawn to allow instant reperfusion. Buprenorphine was given every 6 h on the day of surgery and then supplied *via* the drinking water (1 mg/kg). To encourage eating, the mice received softened chow in a Petri-dish placed on the floor of their cages. Baclofen (25 μg/kg, i.p.) was dissolved in sterile PBS and injected immediately after starting reperfusion. Sham operated mice were kept for 1 h in a recovery box at 37°C and were injected at the same time point as MCAO operated mice. Except for the experiment shown in Figure 3, sham operated mice were sacrificed time matched with MCAO operated mice. Successful MCAO surgery was assessed by testing for impaired neurological/motor function using the Bederson score (Schaar et al., 2010).

**FIGURE 1:**
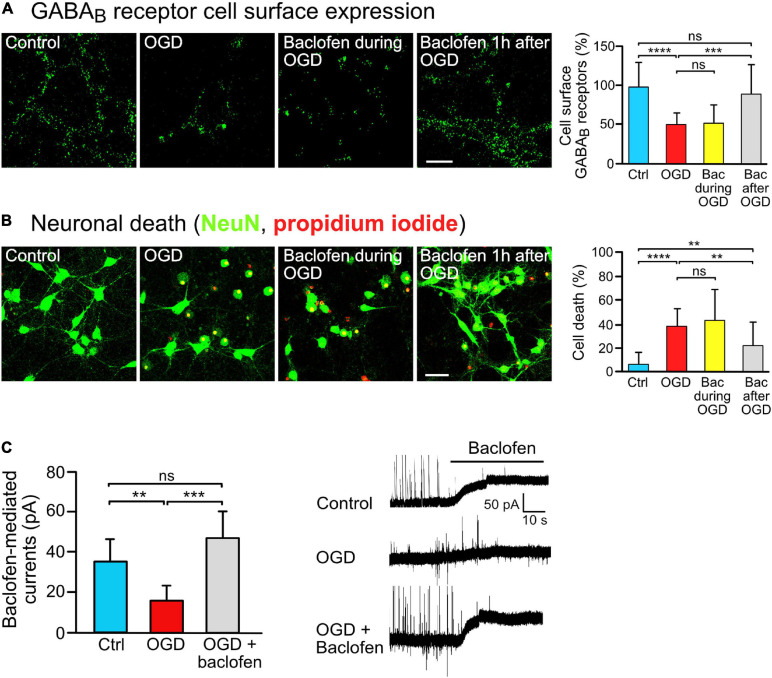
Baclofen treatment restored GABA_*B*_ receptor surface expression and was neuroprotective after OGD stress *in vitro*. **(A)** Baclofen treatment after, but not during, OGD increased GABA_*B*_ receptor cell surface expression. Cultures were treated with baclofen (50 μM) during or after OGD. After 16 h after OGD, neurons were then analyzed for the level of GABA_*B*_ receptor surface expression using antibodies directed against GABA_*B2*_. The incubation time with baclofen was 1 h in the “Bac during OGD” condition and 16 h in the “Bac after OGD” condition. Left, representative images (scale bar, 10 μm). Right: the bar graph depicts the quantification of fluorescence intensities. The data represent the mean ± SD of 18–23 neurons per condition from two independent experiments. One-way ANOVA followed by Tukey’s Multiple Comparison test (ns, *p* > 0.05; ***, *p* < 0.001; ****, *p* < 0.0001). **(B)** Treatment with baclofen after, but not during, OGD reduced neuronal loss *in vitro*. Cultures were treated as in panel **(A)** and neuronal loss was determined using propidium iodide (red) and NeuN staining (green) 24 h after OGD. Left, representative images (scale bar, 40 μm). Right: the bar graph depicts the quantification of fluorescence intensities. The data represent the mean ± SD of 40–41 neurons per condition from three independent experiments. One-way ANOVA followed by Tukey’s Multiple Comparison (ns, *p* > 0.05; **, *p* < 0.01; ****, *p* < 0.0001). **(C)** Baclofen treatment normalized GABA_*B*_ receptor mediated currents in OGD-stressed neurons. Cultured neurons were subjected to OGD and treated or not for 16 h with baclofen. After washing to remove baclofen, baclofen-induced currents were measured for a duration of 10 min using whole cell voltage clamp recordings. Right, representative voltage clamp traces. The data represent the mean ± SD of 6–9 neurons per condition from two independent neuron preparations. One-way ANOVA with Tukey’s Multiple Comparison *post hoc* test (ns, *p* > 0.5; **, *p* < 0.01; ***, *p* < 0.001).

**FIGURE 2:**
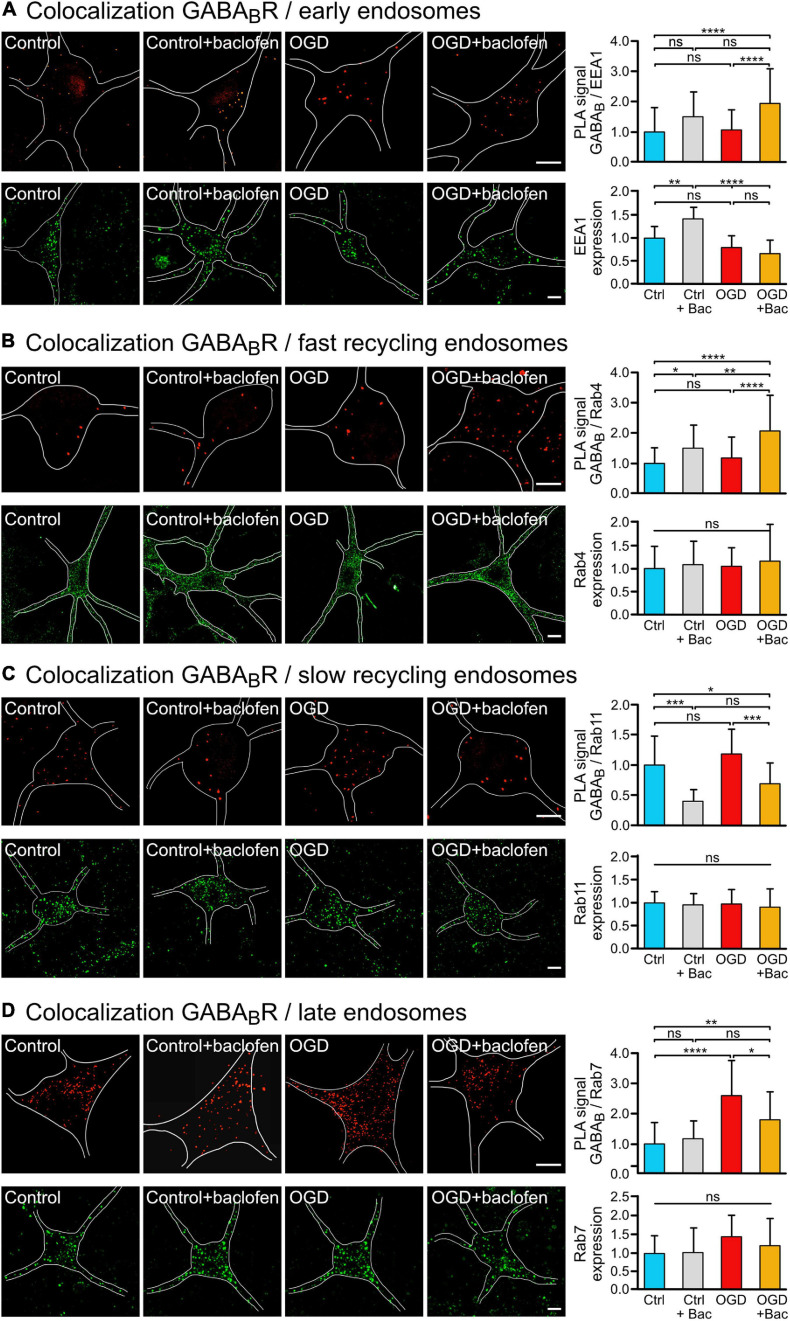
Sustained baclofen stimulation of GABA_*B*_ receptors reduced sorting of the receptors to lysosomal degradation after OGD and increased fast recycling. Cultured neurons were subjected (OGD) or not (Ctrl) to OGD for 1 h followed by incubation with baclofen (+Bac). Neurons were analyzed 16 h after OGD for the colocalization of GABA_*B*_ receptors with endosomal markers by *in situ* PLA (upper panels) and for expression of the marker proteins (lower panels) using antibodies directed against GABA_*B2*_ and EEA1 **(A)**, Rab4 **(B)**, Rab11 **(C)**, and Rab7 **(D)**. **(A)** Baclofen treatment increased the colocalization of GABA_*B2*_ with the early endosome marker EEA1 after OGD. Left, representative images depicting colocalization (upper panels) and EEA1 expression (lower panels) (scale bar, 10 μm). Right: the bar graphs depicts the quantification of PLA signals and fluorescence intensities. The data represent the mean ± SD of 28–68 neurons per condition from thee independent experiments for *in situ* PLA and 14–20 neurons per condition for EEA1 expression. Two-way ANOVA followed by Tukey’s *post hoc* test (ns, *p* > 0.05; **, *p* < 0.005; ****, *p* < 0.0001). **(B)** Baclofen treatment increased the colocalization of GABA_*B2*_ with the fast recycling endosome marker Rab4. Left, representative images (scale bar, 10 μm). Right: the bar graphs depicts the quantification of PLA signals and fluorescence intensities of Rab4 expression. The data represent the mean ± SD of 39–46 neurons per condition from thee independent experiments for *in situ* PLA and 49–70 neurons per condition for Rab4 expression. Two-way ANOVA followed by Tukey’s *post hoc* test (ns, *p* > 0.05; *, *p* < 0.05; **, *p* < 0.01; ****, *p* < 0.0001). **(C)** Baclofen treatment reduced colocalization of GABA_*B*_ receptors with the slow recycling endosome marker Rab11. Left, representative images (scale bar, 10 μm). Right: the bar graphs depicts the quantification of PLA signals and fluorescence intensities of Rab11 expression. The data represent the mean ± SD of 13–26 neurons per condition from three independent experiments for *in situ* PLA and 32–58 neurons per condition for Rab11 expression. Two-way ANOVA followed by Tukey’s *post hoc* test (ns, *p* > 0.05; *, *p* < 0.05; ***, *p* < 0.001). **(D)** Baclofen treatment reduced the OGD-induced colocalization of GABA_*B*_ receptors with the late endosome marker Rab7. Left, representative images (scale bar, 10 μm). Right: the bar graphs depicts the quantification of PLA signals and fluorescence intensities of Rab7 expression. The data represent as mean ± SD of 12–32 neurons per condition from two independent experiments for *in situ* PLA and 16–25 neurons per condition for Rab7 expression. Two-way ANOVA followed by Tukey’s *post hoc* test (ns, *p* < 0.05; *, *p* < 0.05; **, *p* < 0.01; ****, *p* < 0.0001).

**FIGURE 3:**
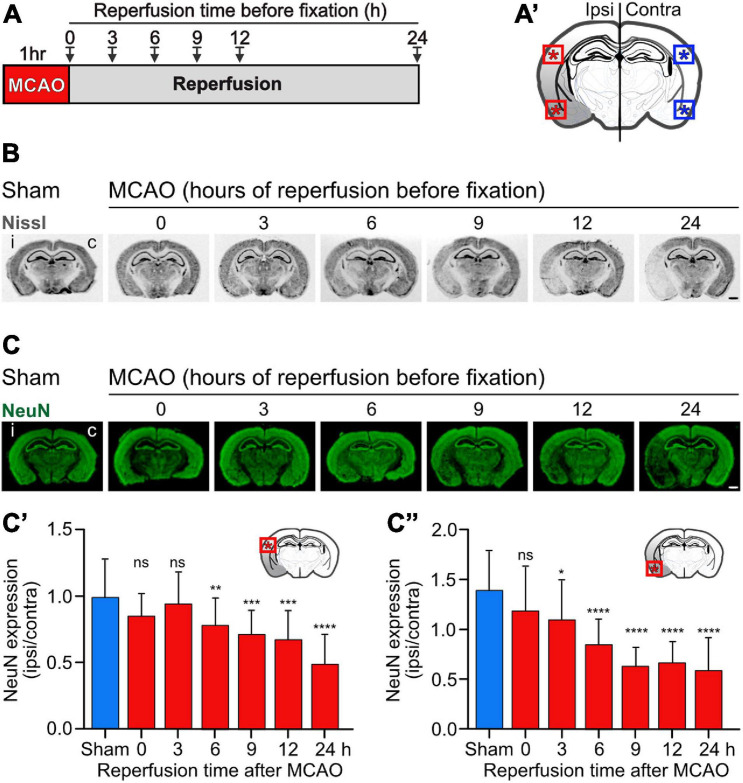
Progressing neuronal loss in the cerebral cortex in the mouse MCAO model of ischemic stroke. Mice were subjected to Sham or MCAO surgery followed by 0, 3, 6, 9, 12, and 24 h reperfusion. Brains were then fixated and analyzed for neuronal loss in the cerebral cortex using Nissl **(B)** and NeuN **(C)** staining. **(A)** Scheme of the experimental design. **(A′)** Schematic drawing of a coronal mouse brain section indicating the areas analyzed in this study (dorsal and ventral cortex). **(B)** Representative images of Nissl stained brain sections obtained from Sham and MCAO treated mice reperfused for the indicated time intervals before fixation (scale bar, 1 mm). **(C)** Representative images of NeuN stained brain sections (scale bar, 1 mm). **(C′)** Quantification of fluorescence intensities of NeuN staining in the dorsal cortex normalized to the fluorescence signals of the unaffected contralateral cortex. **(C″)** Quantification of fluorescence intensities of NeuN staining in the ventral cortex normalized to the fluorescence signals of the unaffected contralateral cortex. The data represent the mean ± SD of 12–25 brain sections obtained from three mice and two independent staining experiments. One-way ANOVA followed by Tukey’s Multiple Comparison *post hoc* test (ns, *p* > 0.05; *, *p* < 0.05; **, *p* < 0.01; ***, *p* < 0.001; ****, *p* < 0.0001).

### Electrophysiology and Acute Brain Slice Preparation

For *ex vivo* electrophysiological recordings, sham or MCAO operated mice were sacrificed 1 h after baclofen or saline injection for slice preparation and recorded *ex vivo* within 3–6 h. Isoflurane anesthetized mice were decapitated and their brains quickly transferred into ice-cold solution containing 65 mM NaCl, 2.5 mM KCl, 1.25 mM NaH_2_PO_4_, 25 mM NaHCO_3_, 7 mM MgCl_2_, 0.5 mM CaCl_2_, 25 mM glucose, and 105 mM sucrose saturated with 95% O_2_ and 5% CO_2_. Coronal sections (300 μm) containing the cortical areas affected by MCAO were sliced using a vibratome (HM 650, Microm). Slices were transferred to a recovery chamber containing oxygenated artificial cerebrospinal fluid (ACSF, 315 mOsm) saturated with 95% O_2_ and 5% CO_2_ and containing 125 mM NaCl, 2.5 mM KCl, 1.25 mM NaH_2_PO_4_, 25 mM NaHCO_3_, 1 mM MgCl_2_, 2 mM CaCl_2_, and 25 mM glucose at 34°C for 25 min and then at room temperature. Following an additional 30 min recovery period, the slices were transferred to a recording chamber perfused with oxygenated ACSF at a flow rate of 1–2 ml/min. All electrophysiology experiments were performed at 32–34°C.

Neurons in the somatosensory cortex to be recorded were visualized by infrared illumination using an Olympus fluorescent microscope (BX51WI). Whole-cell patch-clamp recordings were performed from excitatory neurons, which were identified based on their morphology and firing patterns. The intracellular solution used to measure GABA_*B*_ receptor-mediated GIRK currents and neuronal excitability consisted of 135 mM potassium gluconate, 2 mM NaCl, 4 mM KCl, 4 mM EGTA, 10 mM HEPES pH 7.3, 4 mM Mg-ATP, and 0.3 mM Na_3_GTP and was filled in borosilicate glass patch pipettes (3.5–4.5 mΩ).

Current-clamp experiments were performed using a series of current steps (in 50 pA increments from −350 to 600 pA, 250 ms duration). Neurons were maintained at their original resting membrane potential throughout the experiment. The input resistance was determined using Ohm’s law after calculating the slope of the current–voltage (I–V) curves obtained in current clamp mode. The voltage responses were measured following a series of 50 pA hyperpolarize current pulses, the resting membrane potential (*V*_*m*_) was determined immediately after acquiring the whole cell patch mode and the firing threshold (*V*_*t*__*h*_) was determined by injection of 5 pA depolarization current steps until the first action potential was generated.

For agonist-induced currents, neurons were hold at −50 mV and changes in holding currents in response to bath application of 100 μM baclofen were measured for 15 min. GABA_*B*_ receptor-mediated GIRK currents were confirmed by application of the GABA_*B*_ receptor antagonist CGP36742. For the analysis of GIRK currents in cultured neurons, the cultures were subjected to OGD and treated for 16 h with baclofen. After washing to remove baclofen, baclofen-induced currents were measured for a duration of 10 min.

Series resistance (Rs) was monitored throughout the experiment and recordings were excluded from the analysis if the Rs varied by 25%. Currents were filtered at 5 kHz and digitized at 20 kHz. All recordings were performed using a Multiclamp 700B amplifier, acquired with Digidata 1550A, and analyzed offline using Clampfit 10.5 (Molecular Devices).

### Immunostaining and Image Analysis

#### Staining of Cultured Neurons

##### Total cell staining

Neurons were washed with PBS, fixated with 4% paraformaldehyde (PFA) containing 4% sucrose for 20 min and then permeabilized for 12 min with 0.2% Triton X-100 in PBS. Subsequently, neurons were incubated overnight at 4°C with primary antibody diluted in PBS containing 10% normal goat serum (NGS) or normal donkey serum (NDS) based on the antibody used. After extensive washing with PBS, secondary antibodies were added for 1 h at room temperature. Subsequently, the coverslips were washed with PBS and mounted onto glass slides with DAKO fluorescence mounting medium for analysis by confocal microscopy.

##### Cell surface staining

Cultured neurons were washed with incubation buffer (25 mM HEPES pH 7.4, 119 mM NaCl, 5 mM KCl, 2 mM CaCl_2_, 2 mM MgCl_2_, 30 mM glucose) and incubated with anti-GABA_*B*_ receptor antibodies diluted in incubation buffer containing 10% NDS for 2 h on ice. After washing with ice-cold incubation buffer, the cultures were incubated with secondary antibodies for 1 h on ice, washed again and fixated with 4% PFA containing 4% sucrose for 15 min at room temperature. The coverslips were then mounted onto glass slides with DAKO fluorescence mounting medium for analysis by confocal microscopy.

##### *In situ* proximity ligation assay

Proximity ligation assay was used to monitor the colocalization of GABA_*B*_ receptors with endosomal marker proteins as previously described (Maier et al., 2014; Zemoura et al., 2019). Cells were washed with PBS, fixated with 4% PFA for 20 min at room temperature followed by permeabilization with 0.2% Triton X-100 in PBS for 10 min. The cells were then incubated overnight at 4°C with the appropriate pair of antibodies for GABA_*B*_ receptors and the endosomal marker protein diluted in PBS containing 5% BSA. Subsequently, *in situ* PLA was performed using the Duolink kit (Sigma Aldrich) exactly according to the manufacturer’s instructions.

##### Cell survival assay

Neurons were subjected to OGD with a recovery period of 24 h. Thereafter, cells were washed with incubation buffer and incubated in 7.5 μM propidium iodide solution (Sigma Aldrich) for 25 min at 37°C, 5% CO_2_. Subsequently, cultures were fixated, permeabilized, and stained for neurons using NeuN antibodies. Images were taken with a LSM 510 Meta confocal microscope equipped with a Plan-Neofluar 40×/1.3 DIC oil-immersion objective (Carl Zeiss) and the number of NeuN and propidium iodide positive neurons was determined.

##### Confocal microscopy and quantitative image analysis

Images were recorded with a LSM 510 Meta confocal microscope (Carl Zeiss) equipped with a Plan-Neofluar 40×/1.3 DIC and Plan-Fluar 100×/1.45 DIC oil-immersion objective or with a LSM 800 confocal microscope (Carl Zeiss) using a Plan-Apochromat 40×/1.4 or 63×/1.4 DIC oil-immersion objective. Images containing 4–5 optical sections with 0.4–1.0 μm spacing were recorded at a resolution of 1024 × 1024 pixels and used for the analysis of fluorescence intensities, counting of neurons or the number of fluorescent spots in case of *in situ* PLA. Laser intensities and detector gain were adjusted so that all signals were below saturation. Images were analyzed using the ImageJ software^1^.

For quantification of the cell surface staining, the optical sections of each *z*-stack were merged into one image and the outer and the inner perimeter of the cell surface containing the fluorescence signals were exactly outlined. Then the mean fluorescence intensity value obtained from the inner border was subtracted from the one of the outer border.

For quantification of *in situ* PLA signals, the cell soma was marked carefully to allow particles quantification within the cell using the ImageJ plug-in *Analyze Particles*. Before counting signal dots, a Gaussian blur filter was applied (sigma = 1), followed by background subtraction (Rolling Ball radius = 30). The build-in Moments algorithm was applied to determine the signal threshold for all images. The number of dots representing the PLA signal was then determined.

#### Staining of Brain Sections

##### Immunostaining

**FIGURE 4:**
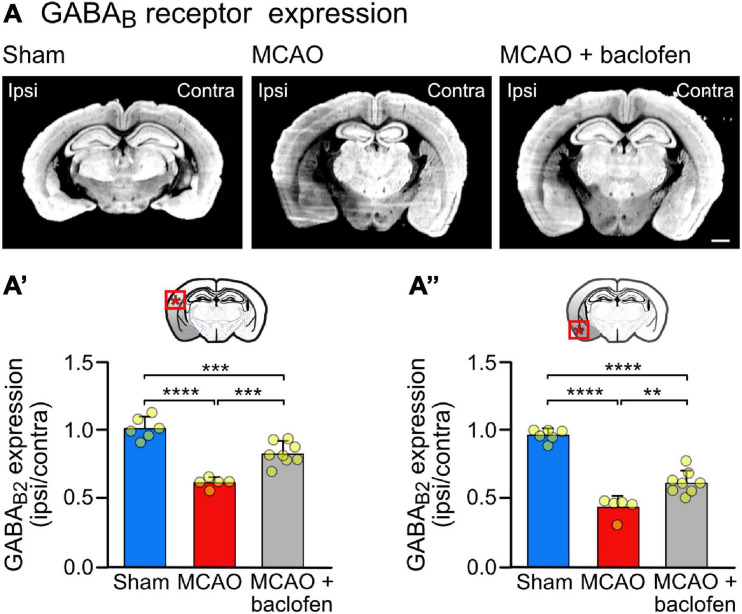
Cerebral ischemia reduces the expression of GABA_*B*_ receptor in the neocortex. Mice were subjected to Sham or MCAO surgery and instantly thereafter injected with baclofen (i.p., 25 mg/kg) or saline, followed by 6 h reperfusion. **(A)** Representative coronal sections depicting the expression of GABA_*B*_ receptors (scale bar, 1 mm). **(A′)** Quantification of the fluorescence intensities in the ipsilateral dorsal cortex normalized to the corresponding contralateral side. **(A″)** Quantification of the fluorescence intensities in the ipsilateral ventral cortex normalized to the corresponding contralateral side. The data represent the mean ± SD of slices/mice obtained from: sham = 43/6; MCAO = 39/5; MCAO + Baclofen = 60/8. The data points represent the average fluorescence intensity derived from all slices analyzed per mouse. One-way ANOVA followed by Tukey’s Multiple Comparison test (**, *p* < 0.01; ***, *p* < 0.001; ****, *p* < 0.0001).

Following either sham or MCAO surgery, mice were anaesthetized with pentobarbital (100 mg/kg, i.p.) and transcardiacally perfused with ice-cold ACSF. Brains were instantly extracted and embedded in NEG50 (Richard-Allan Scientific, Fisher Scientific, Switzerland) and stored at −80°C. For immunostaining, 30 μm sections were cut using a cryostat (Hyrax 60, Carl Zeiss) and mounted onto glass slides (SuperFrost Plus, Thermo Scientific). The sections were fixated using 4% PFA for 30 min at room temperature, followed by antigen retrieval in a PickCell 2100-Retriever in 10 mM citrate buffer pH 6.0, 0.05% Tween 20 for 20 min. Sections were then permeabilized (0.2% Triton X-100 in 10 mM Tris pH 7.4, 150 mM NaCl, 10% NGS) and incubated with primary antibody diluted in TBST (10 mM Tris pH 7.4, 150 mM NaCl, 0.05% Tween-20) containing 10% NGS overnight at 4°C. After washing five times for 5 min with TBST, the sections were incubated with secondary antibodies (Alexa Fluor Plus 800) for 1 h at room temperature. After washing with TBST, sections were air dried in the dark and scanned with an Odyssey CLx infrared scanner (Licor) at a resolution of 21 μm (Figures 4A, 6A) and 42 mm (Figure 3C).

**FIGURE 5:**
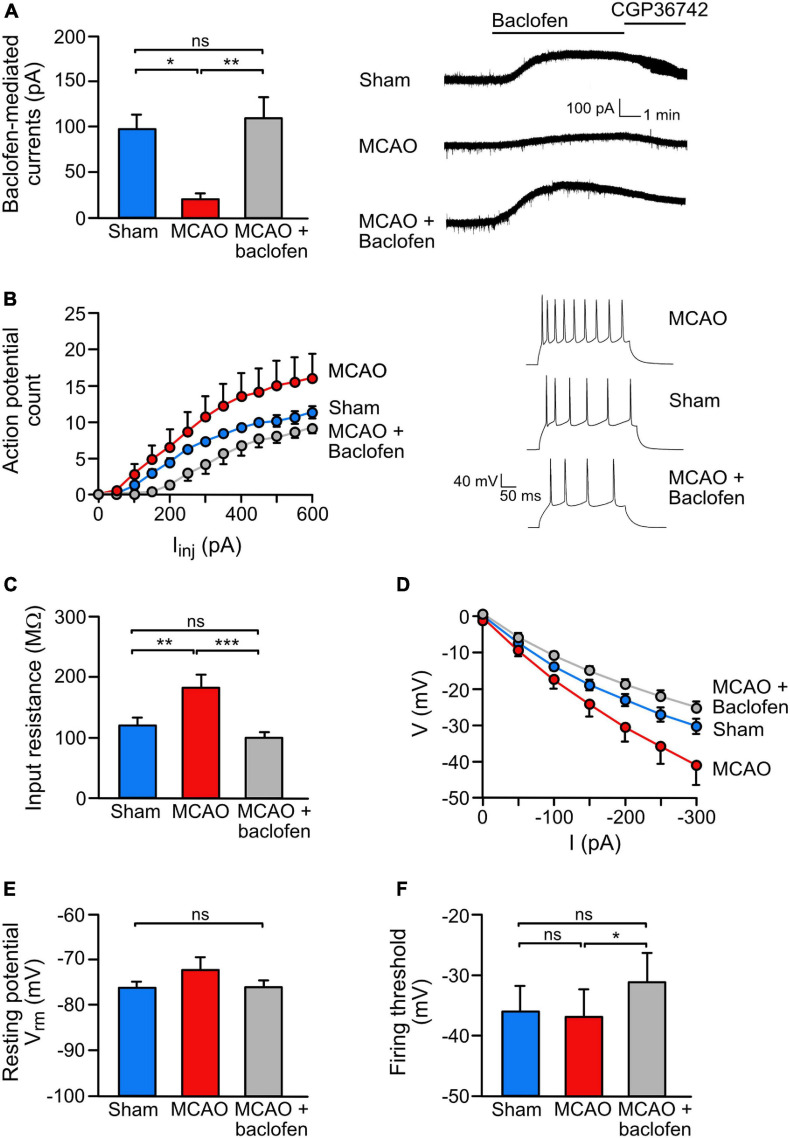
Baclofen treatment rescued the ischemia-induced reduction of GABA_*B*_ receptor-mediated currents and reduced neuronal hyperexcitability. **(A)** Baclofen treatment normalized GABA_*B*_ mediated currents in the ischemic brain. Coronal brain sections obtained from sham or MCAO treated mice were recorded *ex vivo* within 3–6 h after baclofen or saline injection injection. Mice were sacrifized for slice perparation 1 h after baclofen or saline injection. Baclofen-induced currents were measured for a duration of 15 min using whole cell voltage clamp recordings. Right, representative voltage clamp traces. One-way ANOVA with Tukey’s Multiple Comparison *post hoc* test (ns, *p* > 0.5; *, *p* < 0.05; **, *p* < 0.01). **(B)** Baclofen injection reduced ischemia-induced neuronal hyperexcitability. The experimental conditions were the same as in panel **(A)** except that recordings were obtained in the current clamp mode. Neurons of MCAO-treated mice show an increased intrinsic excitability reflected by an increased number of action potentials elicited by a series of depolarizing current pulses (50 pA/250 ms from resting membrane potential). Repeated measures ANOVA with mixed-effects analysis showed a significant interaction among AP counts and treatments [*F*(1.226,135.4) = 35.23, *p* < 0.0001). Right, representative current clamp traces elected by 300 pA current pulse. **(C–F)** Baclofen reduced excitability by modulating intrinsic neuronal properties. **(C)** While MCAO increased the input resistance, treatment with baclofen reduced the values to sham levels. This effect was calculated from the slope of I–V curves **(D)**. One-way ANOVA with Tukey’s Multiple Comparison *post hoc* test (ns, *p* > 0.05; **, *p* < 0.01; ***, *p* < 0.001). **(E)** The analysis revealed no change in the resting membrane potential and no change in the neuronal firing threshold in MCAO mice. However, treatment with baclofen depolarized the action potential firing threshold in MCAO mice. One-way ANOVA with Tukey’s Multiple Comparison *post hoc* test (ns, *p* > 0.05; *, *p* < 0.05). The data represent the mean ± SEM for the number of cells/mice: sham = 19/6, MCAO = 16/5, R1 = 9/4, Rand = 11/5.

**FIGURE 6:**
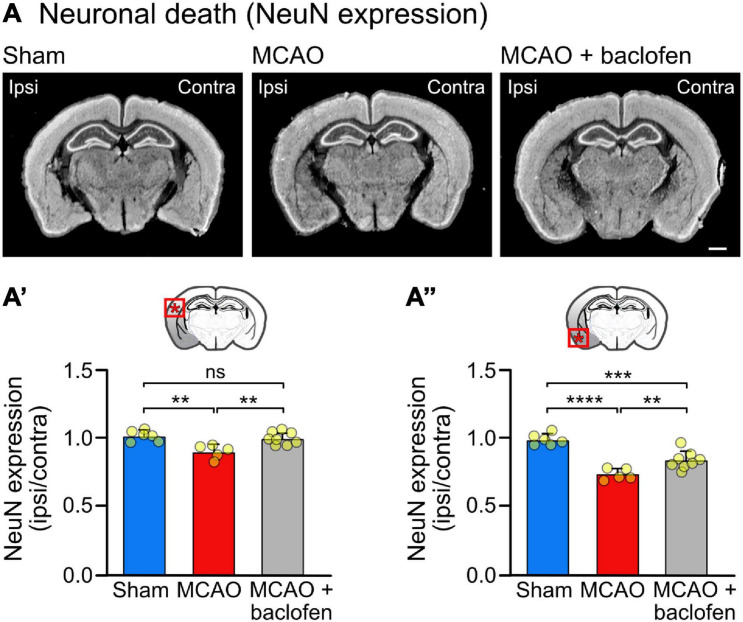
Baclofen treatment after MCAO is neuroprotective. Mice were subjected to MCAO and injected with baclofen or saline followed by 6 h reperfusion. **(A)** Representative images depicting the expression of NeuN (scale bar, 1 mm). **(A′)** Quantification of the fluorescence intensities in the indicated area in the ipsilateral dorsal cortex normalized to the corresponding contralateral side. **(A″)** Quantification of fluorescence intensities in the ipsilateral ventral cortex normalized to the corresponding contralateral side. The data represent the mean ± SD of slices/mice obtained from: sham = 47/6; MCAO = 40/5; MCAO + Baclofen = 61/8). The data points represent the average fluorescence intensity derived from all slices analyzed per mouse. One-way ANOVA followed by Tukey’s Multiple Comparison test (ns, *p* > 0.05; **, *p* < 0.01; ***, *p* < 0.001; ****, *p* < 0.0001).

##### Nissl staining

Paraformaldehyde fixated and permeabilized sections were incubated for 15 min in 0.5% cresyl violet solution, washed for 30 s in H_2_O and destained in ethanol for 20 s up to 2 min. The sections were then dehydrated by a series of isopropanol, isopropanol/xylene and xylene incubations and finally coversliped in Eukitt (Sigma Aldrich).

### Statistical Analysis

Data represent means ± SD except for electrophysiological results which are indicated as mean ± SEM for better display. Statistical comparisons were made with either one-way or two-way analysis of variance (ANOVA) followed by Tukey’s *post hoc* tests. Statistical tests were performed using GraphPad Prism 8. Differences were considered statistically significant when *p* < 0.05.

## Results

### Baclofen Treatment After Excitotoxic Stress *in vitro* Restored Cell Surface Expression of GABA_*B*_ Receptors

To investigate the impact of sustained baclofen activation of GABA_*B*_ receptors on cell surface expression of the receptors under ischemic/excitotoxic conditions, we utilized OGD of primary cortical neurons as an *in vitro* model. Consistent with previous research (Guetg et al., 2010; Maier et al., 2010, 2014; Terunuma et al., 2010), exposing neurons to excitotoxic conditions considerably reduced cell surface expression of GABA_*B*_ receptors (Figure 1A). To tested whether sustained stimulation of GABA_*B*_ receptors affects receptor surface expression following OGD, we administered baclofen during OGD or thereafter and determined cell surface expression of the receptors after a recovery period of 16 h. Baclofen treatment only during OGD did not affect cell surface GABA_*B*_ receptor expression, whereas application of baclofen after OGD restored receptor expression close to control levels (Figure 1A). Under these conditions, baclofen treatment only during OGD did not affect loss of neurons tested 24 h after OGD, whereas treatment after OGD significantly reduced loss of neurons (Figure 1B). The ineffectiveness of baclofen treatment when applied only during OGD might be due to an inappropriate timing of the application or to a too short duration of the application.

Finally, we tested whether baclofen treatment restored GABA_*B*_ receptor function as measured by baclofen-induced K^+^ currents. OGD strongly reduced baclofen evoked K^+^ currents in neurons as compared to control neurons (Figure 1C), which is in line with the considerable downregulation of the receptors observed in Figure 1A. Treatment of OGD-stressed neurons with baclofen immediately after OGD for 16 h restored GABA_*B*_ receptor-mediated currents to a level of unstressed control neurons (Figure 1C).

To explore the pathway underlying the baclofen-induced restoration of cell surface GABA_*B*_ receptors after OGD, we analyzed the co-localization of the receptors with marker proteins for early endosomes (EEA1) (Mu et al., 1995; Wilson et al., 2000), fast recycling endosomes (Rab4) (van der Sluijs et al., 1992; Daro et al., 1996), slow recycling endosomes (Rab11) (Ullrich et al., 1996) and late endosomes (Rab7) (Feng et al., 1995) using the *in situ* PLA. OGD did not affect the colocalization of GABA_*B*_ receptors with EEA1 (Figure 2A), Rab4 positive fast recycling endosomes (Figure 2B) or Rab11 positive slow recycling endosomes (Figure 2C). However, baclofen treatment increased the colocalization of GABA_*B*_ receptors with Rab4 under control condition and after OGD (Figures 2A,B). In contrast, baclofen treatment strongly reduced the co-localization of the receptors with Rab11 in control as well as in OGD treated neurons (Figure 2C). This indicates a switch from slow recycling Rab11 positive perinuclear endosomes to fast recycling of the receptors from Rab4 positive endosomes. Finally, OGD considerably increased the colocalization of GABA_*B*_ receptors with Rab7, indicating increased sorting to the lysosomal degradation pathway (Figure 2D). Treatment with baclofen significantly reduced the colocalization with Rab7 after OGD, whereas under control conditions baclofen did not affect the colocalization with Rab7 (Figure 2D). The expression levels of endosomal marker proteins were not changed by the treatments except for EEA1 (Figure 2A). Baclofen treatment increased EEA1 expression in control neurons but not in OGD stressed neurons. Hence, the trend of enhanced colocalization of EEA1 and GABA_*B*_ receptors observed in baclofen-treated control neurons (Figure 2A) might be caused by the upregulation of EEA1.

These findings suggest that sustained activation of GABA_*B*_ receptors after an ischemic insult reduces sorting of GABA_*B*_ receptors to lysosomal degradation by increasing fast recycling on the expense of slow recycling.

### Baclofen Treatment Partially Restored Downregulated GABA_*B*_ Receptor Expression After Cerebral Ischemia *in vivo*

To assess the effect of baclofen on the expression of GABA_*B*_ receptors in cerebral ischemia, we utilized the MCAO mouse model of stroke. First, we determined the tempo-spatial development of loss of neurons, to select suitable conditions for the following experiments. Mice were subjected to 1 h of MCAO and loss of neurons was determined in the neocortex at different time points following reperfusion using Nissl and NeuN staining (Figure 3A). Using Nissl staining, loss of neurons became visible on a macroscopic level 3 h after re-establishing reperfusion in the striatum and ventral cortex. It then spreads with time to dorsal areas of the cortex (Figure 3B). Quantification of neuronal loss using NeuN staining clearly showed progressing neuronal death in the ventral and dorsal cortex, reaching severe levels in the dorsal cortex considerably later than in the ventral cortex (Figure 3C). Small but significant levels of neuronal loss were present in the ventral and dorsal cortex 6 h after reperfusion (Figure 3C). We used this time point to investigate the impact of baclofen on the expression and function of GABA_*B*_ receptors to minimize the effect of neuronal loss in the following experiments. At this timepoint, we observed a significant reduction in GABA_*B*_ receptor expression in the affected ipsilateral side, in both the dorsal and ventral cortex, as compared to sham operated animals (Figure 4). The magnitude of receptor downregulation exceeded by far the low level of neuronal loss at this time point and, therefore, could not be explained by a reduced number of neurons. Receptor expression was partially recovered following intraperitoneal injection of baclofen immediately after MCAO (Figure 4). Thus, agonist stimulation of GABA_*B*_ receptors after cerebral ischemia partially restored GABA_*B*_ receptor expression *in vivo*.

### Baclofen Treatment After MCAO Restored GABA_*B*_ Receptor-Mediated Inhibition and Reduced Neuronal Excitability

Next, we analyzed if baclofen treatment after MCAO also restored GABA_*B*_ receptor-mediated inhibition. GABA_*B*_-receptor mediated currents were measured in the dorsal cortex 3–6 h after starting reperfusion using *ex vivo* patch clamp recordings in acute brain slices obtained from mice subjected to MCAO. In line with the immunohistochemical data, we observed a strong reduction of GABA_*B*_ receptor-mediated currents in neurons from MCAO-treated mice as compared to sham operated mice (Figure 5A). Injection of baclofen immediately after starting reperfusion restored GABA_*B*_ receptor-mediated currents to a level of sham operated mice (Figure 5A).

We then tested if the restored GABA_*B*_ receptor-mediated inhibition upon baclofen injection affects neuronal excitability. Compared to sham operated mice, MCAO-treated mice displayed a considerably higher neuronal excitability, as assessed by the number of action potentials elicited by a series of depolarizing current pulses. Injection of baclofen after MCAO, strongly reduced the excitability to the level of sham operated mice (Figure 5B).

To better understand the underlying mechanism by which baclofen reduces neuronal excitability after cerebral ischemia, we measured passive properties of the neurons. After MCAO, the membrane conductance was decreased, as reflected by an enhanced input resistance (Figure 5C) and a shift in the I–V curve (Figure 5D), indicating increased excitability of the neurons. Injection of baclofen normalized the input resistance and the shifted IV curve back to levels of sham operated mice (Figures 5C,D). Furthermore, we did not observe a significant difference in neuronal resting potential or firing threshold after MCAO (Figures 5E,F). However, administration of baclofen depolarized the firing threshold (Figure 5F), which is known to contribute to the reduction on firing frequency observed in the baclofen injected mice (Deng et al., 2009).

These results indicate that administration of baclofen directly after re-establishing reperfusion in MCAO-treated mice restored downregulated GABA_*B*_ receptor-mediated inhibition and reduced elevated excitability of neurons.

### Baclofen Treatment Following Cerebral Ischemia Reduced Neuronal Loss

To test the neuroprotective activity of the baclofen treatment, we quantified NeuN staining in brain sections derived from MCAO operated mice treated with baclofen or saline immediately after starting reperfusion and sacrificed 6 h thereafter (Figure 6). In line with the experiments shown in Figure 3, we observed a small but significant level of neuronal loss at this early time point in the dorsal cortex, which was prevented by treatment with baclofen (Figure 6A′). In the more severely affected ventral cortex baclofen likewise exhibited a neuroprotective effect (Figure 6A″). These results indicate that baclofen treatment effectively promotes survival of cortical neurons.

## Discussion

Here we show that sustained activation of GABA_*B*_ receptors after an ischemic insult restores downregulated GABA_*B*_ receptor expression and function, which limited excitotoxic neuronal death. This study is based on previous work demonstrating that GABA_*B*_ receptors are severely downregulated under excitotoxic conditions (Guetg et al., 2010; Maier et al., 2010, 2014; Terunuma et al., 2010; Kim et al., 2011; Kantamneni et al., 2014; Zhu et al., 2015; Huang et al., 2017) and indications from work on GABA_*B*_ receptors recombinantly expressed in HEK 293 cells that sustained activation of the receptors with baclofen stabilized receptor expression on the cell surface (Zhang et al., 2015). We hypothesized that treatment with baclofen after an ischemic insult may reduce downregulation of the receptors and re-establish GABA_*B*_ receptor-mediated inhibition. This would explain the well-established neuroprotective activity of baclofen (Lal et al., 1995; Jackson-Friedman et al., 1997; Kulinskii and Mikhel’son, 2000; Babcock et al., 2002; Dave et al., 2005; Zhang et al., 2007; Xu et al., 2008; Cimarosti et al., 2009; Liu et al., 2015) although the receptors are severely downregulated under excitotoxic conditions.

Our *in vitro* experiments on cultured neurons subjected to OGD, to mimic cerebral ischemia, verified the strong downregulation of GABA_*B*_ receptors reported previously under excitotoxic conditions provoked by prolonged exposure to NMDA or glutamate (Guetg et al., 2010; Maier et al., 2010; Terunuma et al., 2010; Kantamneni et al., 2014). Treatment of neurons with baclofen after OGD stress indeed recovered GABA_*B*_ receptor expression at the cell surface by the mechanism proposed by Zhang et al. (2015) based on their finding on receptors expressed in HEK 293 cells. Interestingly, baclofen treatment after OGD restored downregulated receptor expression close to normal levels. This finding indicates that under ischemic conditions the supply of new receptors to the cell surface, their internalization, recycling, and degradation mechanisms are still in operation but with altered rates. Our colocalization experiments with endosomal marker proteins for the different intracellular trafficking pathways indicate OGD-enhanced sorting of GABA_*B*_ receptors to the lysosomal degradation pathway, which is in line with previous reports using NMDA and glutamate induced excitotoxicity (Maier et al., 2010; Terunuma et al., 2010; Zemoura et al., 2019). Our results show that baclofen treatment after OGD restores cell surface receptor expression by increasing internalization (as indicated by enhanced colocalization with EEA1 positive endosomes) and fast recycling *via* Rab4 positive endosomes on the expense of slow recycling *via* Rab11 positive endosomes. This finding is in accordance with the observation that disruption of GABA_*B*_ receptor recycling increased lysosomal degradation of the receptors (Grampp et al., 2008; Maier et al., 2010; Zhang et al., 2015). Hence, our experimental data supports a mechanism in which sustained activation of GABA_*B*_ receptors by baclofen induces fast recycling of the receptors to bypass sorting of the receptors to the lysosomal degradation pathway.

Employing the MCAO mouse model of cerebral ischemia, we confirmed progressing neuronal loss and diminished GABA_*B*_ receptor expression in the affected cerebral cortex, which were partially restored after injection of baclofen directly after the ischemic insult. Thus, these data recapitulate our *in vitro* findings on cultured neurons subjected to OGD and verified OGD of cultured neurons as valuable *in vitro* model for the analysis of molecular mechanisms involved in cerebral ischemia. An upregulation of GABA_*B*_ receptor expression in animal models of ischemia upon various treatments, including baclofen (Lu et al., 2016), ferulic acid (Cheng et al., 2010), mild hypothermia (Kim et al., 2011), and opposing needling/acupuncture (Xu et al., 2014; Jiang et al., 2016), had been reported, suggesting that upregulated GABA_*B*_ receptor expression contributed to the neuroprotective effect of the treatments.

As expected, restoration of GABA_*B*_ receptors also recovered the severely compromised GABA_*B*_ receptor-mediated inhibition. Although receptor expression was not fully recovered, baclofen treatment restored GABA_*B*_ receptor-mediated currents and lowered enhanced neuronal excitability to levels of sham operated mice. This was sufficient to inhibit ischemia-induced progressing neuronal loss in the less affected dorsal cortex at the time point of testing (6 h after starting reperfusion), whereas there was a smaller neuroprotective effect of baclofen in the more severely affected ventral cortex. This result was expected since it is very unlikely that any neuroprotective treatment, even when given immediately after the ischemic insult, can prevent the almost instantly occurring neuronal damage in the ischemic core.

One important advantage of targeting GABA_*B*_ receptors for neuroprotection lays in their regulation of multiple beneficial pathways. The most obvious one is the inhibition of glutamate release (Ouyang et al., 2007), reducing the detrimental overstimulation of all members of the glutamate receptor family, and the activation of postsynaptic GABA_*B*_ receptors, which inhibits neuronal excitability (Deng et al., 2009; Chalifoux and Carter, 2011a, b). These combined effects reduce Ca^2+^ overload of the neurons and inhibit multiple mechanisms of glutamate receptor and Ca^2+^ triggered apoptotic neuronal death. In addition to inhibiting neuronal activity, sustained GABA_*B*_ receptor activity also promotes neuronal survival *via* the PI3K/Akt-GSK3β pathway (Tu et al., 2010; Liu et al., 2015; Fu et al., 2016), which is a major neuronal survival cascade (Rai et al., 2019). Finally, baclofen treatment inhibits the upregulation of the pro-apoptotic transcription factor CHOP, which induces apoptotic cell death in response to ER-stress associated with cerebral ischemia (Fu et al., 2016). These combined effects make GABA_*B*_ receptors a promising target for limiting progressing neuronal death in acute cerebral ischemia.

Clinically, baclofen is effective for the treatment of post-ischemic stroke symptoms, i.e., for the relief of severe spasticity and associated pain (Taira et al., 1994; Meythaler et al., 2001; Ivanhoe et al., 2006; Kofler et al., 2009; Creamer et al., 2018a, b) and persistent hic-ups (Zhang et al., 2014). There is also indication from animal studies for a potential benefit for the treatment of anxiety (Lu et al., 2016) and sleep disturbances (Hodor et al., 2014) associated with cerebral ischemia. However, the neuroprotective activity of baclofen in acute stroke remains to be established clinically. Considering the multiple beneficial pathways targeted, sustained activation of GABA_*B*_ receptors might be a promising component for a therapy aimed at limiting progressing excitotoxic neuronal death after an ischemic insult.

## Data Availability Statement

The original contributions presented in the study are included in the article/supplementary material, further inquiries can be directed to the corresponding author.

## Ethics Statement

The animal study was reviewed and approved by Cantonal Veterinary Office Zurich, Zurich, Switzerland (license ZH152/16 and ZH011/19).

## Author Contributions

MH, MV, MB, and KB performed the experimental work and analyzed the data. MH and DB designed the study and wrote the manuscript. DB supervised the work and analyzed the data. All authors discussed the results and commented on the manuscript.

## Conflict of Interest

The authors declare that the research was conducted in the absence of any commercial or financial relationships that could be construed as a potential conflict of interest.

## Publisher’s Note

All claims expressed in this article are solely those of the authors and do not necessarily represent those of their affiliated organizations, or those of the publisher, the editors and the reviewers. Any product that may be evaluated in this article, or claim that may be made by its manufacturer, is not guaranteed or endorsed by the publisher.

## Footnotes

1
https://imagej.nih.gov/ij
